# The phase diagram of a mixed halide (Br, I) hybrid perovskite obtained by synchrotron X-ray diffraction†

**DOI:** 10.1039/c8ra09398a

**Published:** 2019-04-09

**Authors:** Frederike Lehmann, Alexandra Franz, Daniel M. Többens, Sergej Levcenco, Thomas Unold, Andreas Taubert, Susan Schorr

**Affiliations:** Helmholtz-Zentrum Berlin für Materialien und Energie Hahn-Meitner Platz 1 14109 Berlin Germany susan.schorr@helmholtz-berlin.de; University of Potsdam, Institute of Chemistry Karl-Liebknecht-Straße 24-25 14476 Potsdam OT Golm Germany; Free University Berlin, Department of Geoscience Malteserstr. 74-100 12249 Berlin Germany

## Abstract

By using synchrotron X-ray powder diffraction, the temperature dependent phase diagram of the hybrid perovskite tri-halide compounds, methyl ammonium lead iodide (MAPbI_3_, MA^+^ = CH_3_NH_3_^+^) and methyl ammonium lead bromide (MAPbBr_3_), as well as of their solid solutions, has been established. The existence of a large miscibility gap between 0.29 ≤ *x* ≤ 0.92 (±0.02) for the MAPb(I_1−*x*_Br_*x*_)_3_ solid solution has been proven. A systematic study of the lattice parameters for the solid solution series at room temperature revealed distinct deviations from Vegard's law. Furthermore, temperature dependent measurements showed that a strong temperature dependency of lattice parameters from the composition is present for iodine rich compositions. In contrast, the bromine rich compositions show an unusually low dependency of the phase transition temperature from the degree of substitution.

## Abstract 2

The phase diagram elucidates structural changes and phase separation effects, induced by halide substitution in hybrid perovskite MAPb(I,Br)_3_ solid solution.

## Graphical abstract

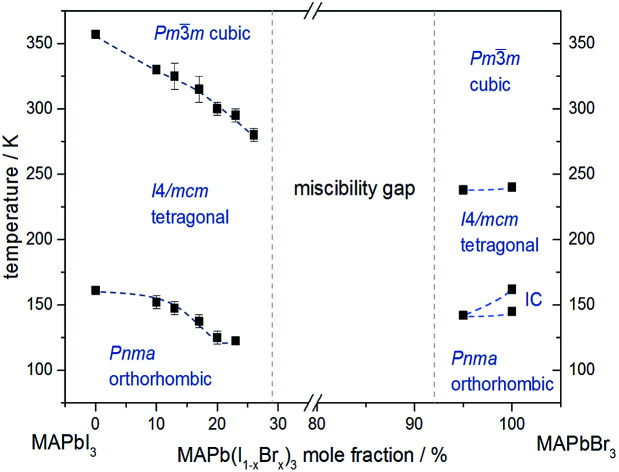

## 1. Introduction

Recently, inorganic–organic – so called “hybrid” – perovskites like methyl ammonium lead iodide (CH_3_NH_3_PbI_3_) have gained a lot of interest as absorber materials for solar cells.^1,2^ Much attention has been focused on their remarkable optoelectronic properties. For instance, their high absorption coefficient^3^ and superior charge carrier mobility^4^ together with the direct bandgap make the material very suitable as an absorber layer in devices with high solar power conversion efficiencies^5–7^ so far exceeding 22.7%.^8^ Additionally, perovskite solar cells are solution-processed, which compared to other photovoltaic technologies is a cost-effective alternative for photovoltaic device fabrication.^5,9^ As the bandgap is directly related to the chemical composition of the semiconductor, bandgap tuning is possible by using solid solutions, offering tailor-made devices. By mixing the halides, the bandgap of CH_3_NH_3_PbX_3_ can be easily tuned over a broad range.^10^ Specifically, the bandgap of CH_3_NH_3_Pb(I_1−*x*_Br_*x*_)_3_ can be continuously adjusted from 1.6 to 2.3 eV, as reported earlier by the group of Noh *et al.*^11^ As a result, hybrid perovskites are very promising low-cost alternatives for application in tandem or multijunction photovoltaics.^12^ Even though the long-term stability and durable outdoor operation are still a big struggle, the commercialization of perovskite solar cells compared to commercial silicon or thin film solar cells is still a topic of intense interest in photovoltaics research. Today perovskite materials are commonly used in diverse energy applications, for instance as semiconductor nanowires for miniature lasers or for smart windows, which can be reversibly tuned into opaque perovskite solar cells using photothermal heating by sunlight. Another useful application of perovskite semiconductors is the use as color-selective photodiodes for color imaging, replacing silicon-based photodiodes and making the use of complex fabricated color filters redundant.^13–16^

Hybrid perovskites have an ABX_3_ composition (here is A = methyl ammonium [CH_3_NH_3_]^+^, B = lead [Pb]^2+^ and X = halide [I, Br]^−^), with A in cuboctahedral coordination of 12X anions and B coordinated by 6X anions, forming a framework of corner-linked PbX_6_ octahedra.^17^ Methyl ammonium lead halide (methyl ammonium abbreviated as MA), similar to other hybrid perovskites, exhibits an orientational disorder of the molecule. Structural investigations on MAPbI_3_ have revealed the existence of three temperature dependent structural modifications: at high temperatures (above 327 K ^18^) the system adopts a cubic phase (*Pm*3̄*m*)^17^ and the methyl ammonium molecule is fully disordered. Between 327 K and 162 K ^18^ the structure is tetragonal (*I*4/*mcm*) and the number of disordered states of the methyl ammonium molecule is lowered to 8 possible orientations,^19^ while at temperatures below 162 K MAPbI_3_ crystallizes in the orthorhombic crystal system with space group *Pnma*, where the orientation of the organic molecule is fixed and appears to be fully ordered.^20^

MAPbBr_3_ crystallizes in the same space groups as MAPbI_3_ with cubic to tetragonal phase transition at 235 K and the tetragonal to orthorhombic phase transition at 148 K.^21^ In addition, an incommensurate phase (IC) has been reported between 148 and 154 K.^18,22^ The substitution of halides additionally influences the ordering and leads to numerous structural phases and phase transitions.^18,22–24^

The current knowledge about MAPb(I_1−*x*_Br_*x*_)_3_ solid solutions is based on several studies on thin films, suggesting different synthesis procedures and their influence on the performance of resulting solar cells. In 2013 the group of Noh *et al.* produced inorganic–organic heterojunction solar cells using the entire range of MAPb(I_1−*x*_Br_*x*_)_3_ to cover an onset absorption band from 1.58 eV to 2.28 eV.^11^ Further investigations on the bandgap tunability of I–Br mixed-halide perovskite films were done by the group of Kulkarni *et al.*, showing that by using a sequential deposition method and varying the concentration of halide precursors, the optical properties of perovskite films can be flexibly modified.^25^ Moreover, Fedeli *et al.* used a single-step solution process and investigated the influence of thermal treatments on the properties of mixed halide (I, Br) perovskite thin films.^26^ Investigations on the optoelectronic properties gained a lot of interest, for instance the group of Hoke *et al.* reported on the formation of a new low energy photoluminescence feature upon light soaking of MAPb(I_1−*x*_Br_*x*_)_3_ perovskites due to photo-induced halide segregation. In MAPb(I_1−*x*_Br_*x*_)_3_ solar cells this is causing a decrease in the electronic bandgap, thus reducing their achievable open circuit voltages.^27^ All in all, numerous investigations have been done on tuning the bandgap and enhancing the efficiency of these mixed halide perovskite solar cells.

As there are still a few information given about the crystal structure of the MAPb(I_1−*x*_Br_*x*_)_3_ solid solutions and the influence of the chemical composition on the phase transition temperatures, a systematic temperature dependent investigation is desired. Analyzing powder or bulk material of MAPb(I_1−*x*_Br_*x*_)_3_ solid solution suggests more potential. For the purpose of optimizing physical properties, it is necessary to understand the structural details of this material. Therefore, the aim of this work is a systematic analysis of the MAPb(I_1−*x*_Br_*x*_)_3_ solid solution over the complete temperature-range from 30 to 350 K by means of direct determination of phase transitions and structural parameters from X-ray power diffraction.

## 2. Experimental

### 2.1 Synthesis

MAPb(I_1−*x*_Br_*x*_)_3_-powder samples were synthesized using a method adapted from Im *et al.*^2^ All reactions were performed in a glovebox under nitrogen atmosphere. MAPbI_3_ was synthesized from stoichiometric mixtures of MAI (99.99% from Ossila) and PbI_2_ (99.999%, metal basis, from Alfa Aesar) in a 1 molar solution of γ-butyrolactone (GBL, ≥99%, Roth) and homogenized overnight at 60 °C. The iodine rich solid solutions (for *x* ≤ 0.3) were synthesized from stoichiometric mixtures of MAI, MABr (99.99% from Ossila) and PbI_2_ in a 1 molar solution of γ-butyrolactone and dimethylformamide (DMF, 99,8%, Roth) (4 : 1 ratio). The solvent was evaporated at 100 °C for MAPbI_3_ and for the iodine rich solid solutions (*x* ≤ 0.3).

For the composition between 0.3 < *x* ≤ 0.6 a stoichiometric mixture of MAI, MABr, PbI_2_ and PbBr_2_ (98+% from Acros Organics) was used in a 1 molar solvent mixture of GBL : DMF (1 : 1 ratio) and an evaporation temperature of the solvent at 100 °C.

MAPbBr_3_ was synthesized from stoichiometric mixtures of MABr and PbBr_2_ in a 1 molar solution of dimethylformamide and homogenized overnight at room temperature. The bromine rich solid solutions (from *x* > 0.6) were synthesized from stoichiometric mixtures of MABr, MAI and PbBr_2_ in a 1 molar solution of γ-butyrolactone and dimethylformamide (1 : 4 ratio). The solvent was evaporated at 80 °C for MAPbBr_3_ and for the bromine rich solid solutions (*x* > 0.6).

Typically, black MAPbI_3_ crystals of isometric habit as well as orange MAPbBr_3_ cube-shaped crystals resulted. The synthesis of MAPb(I_1−*x*_Br_*x*_)_3_ solid solution resulted in polycrystalline samples, varying in colors of black to orange. The use of different solvents (GBL *vs.* DMF) was necessary because hybrid perovskites show a retrograde solubility behavior. First studies on this were performed by the group of Saidaminov *et al.*,^28^ who systematically analyzed the solubility of MAPbI_3_ and MAPbBr_3_ in various solvents at different temperatures, proposing GBL as a suitable solvent for I-based perovskites and the more polar DMF as a suitable solvent for Br-based perovskites. The samples were stored under N_2_ to avoid potential degradation by oxygen and humidity.

### 2.2 Crystallographic characterisation

#### Powder X-ray diffraction

Ground powder samples were analyzed by X-ray powder diffraction at ambient conditions at 293 K. The measurements were performed using a Bruker D8 powder diffractometer with sample spinner (Bragg–Brentano geometry, Cu Kα_1_ radiation (*λ* = 1.5406 Å), step size 0.02, 2*θ* from 10–80°). To receive precise lattice parameters LaB_6_ SRM 660c was added as internal standard to each sample of the iodine rich side (30 wt%) and silicon SRM 640a (30 wt%) to the bromine rich samples, respectively. The data were analyzed by both Le Bail profile fitting and Rietveld analysis using FullProf 2.05.^29^ The instrumental resolution was determined experimentally from a LaB_6_ sample. Peak shape was refined using Thompson–Cox–Hastings pseudo-Voigt function, two angular-dependent asymmetry parameters and *hkl*-dependent Lorentzian broadening described by symmetry adapted spherical harmonics up to 4^th^ order. In Rietveld refinements, for tetragonal phases the model of Franz *et al.*^19^ was used, refining only the anion position and a common Br/I-ratio for both sites. In cubic phases methyl ammonium was modelled as a spherical atom with the same X-ray scattering power (*K*) for simplicity,^30^ refining only the MA Debye–Waller factor and the Br/I-ratio of the anion site. In addition, for each phase independent overall Debye–Waller factors were refined. Lattice parameters derived from Le Bail refinement or from Rietveld are in very good agreement. Those from Le Bail profile fitting were used for discussion.

#### Synchrotron X-ray diffraction

Temperature-dependent synchrotron X-ray diffraction data were collected at KMC-2 beamline^31,32^ at the synchrotron source BESSY II (HZB, Berlin, Germany). The data were collected at a photon energy of 8048 eV, corresponding to Cu Kα_1_ radiation.

For the low-temperature experiments the station was equipped with a modified Gifford–McMahon (GM) closed-cycle cryocooler, in house label CCR-XRD, which – configured with a double Kapton cupola and helium exchange gas – provides very low temperature gradients (see ESI Fig. S3† for detailed descriptions). Due to technical restrictions a fixed incidence angle of 16° was chosen at the sample in reflection geometry. Rotating the sample for increased particle statistics inside the cryostat is not possible, but a minor problem due to the large acceptance angle of the Vantec 2000 detector used. To ensure correct peak positions in the presence of thermal expansion of the sample environment, internal standards for the ambient experiments were used as described above. Data collection series with high temperature resolution (*T* = 30–300 K, Δ*T* = 1 K) for the determination of phase transition temperatures were collected with fixed detector position. For iodine rich samples the observed diffraction angle range was selected around the 220 and 004 Bragg peaks in a 2*θ*-range of 24–34° and the 200 Bragg peak for bromine rich samples in a 2*θ*-range of 28–36.5°, respectively. Full diffractograms (2*θ* = 12.75–93.0°) were taken at selected temperatures: for the iodine rich samples at 30, 200, 298 and 350 K and for bromine rich samples at 30 and 289 K. Total data collection time for one sample was 24 hours to allow for sufficient equilibration.

### 2.3 Photoluminescence

The precise determination of the optoelectronical properties of the MAPb(I_1−*x*_Br_*x*_)_3_ solid solutions was done by photoluminescence measurements on each sample of the composition. The measurements were performed using a 409 nm diode-laser and a thermoelectrically cooled PIXIS100 CCD coupled to ½ grating monochromator. To adjust the excitation density a neutral density filter set was applied.

## 3. Results and discussion

**Fig. 1:**
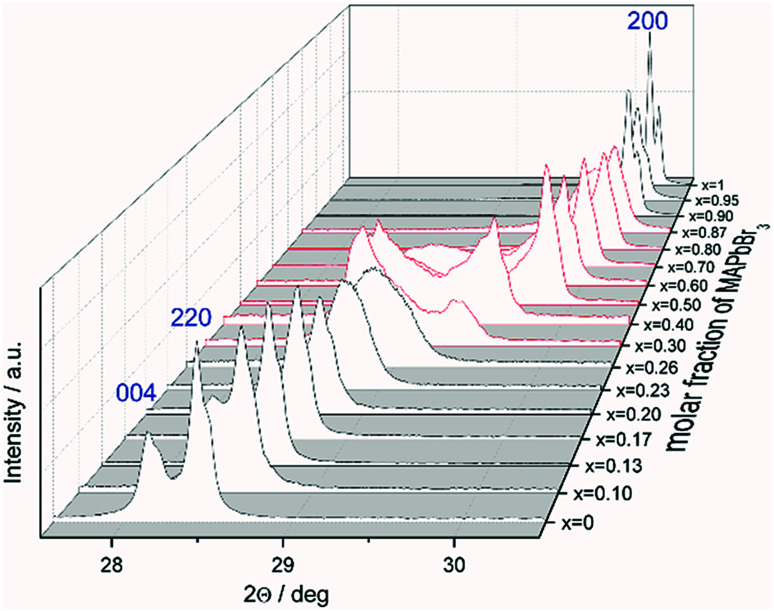
Powder pattern of selected compositions of the MAPb(I_1−*x*_Br_*x*_)_3_ solid solution: evolution of the 220 and 004 Bragg peaks of MAPbI_3_ with increasing degree of substitution. The change from iodine rich phase towards the 200 Bragg peak of the bromine rich phase is shown (single phase region in black, two-phase region in red).

The favored difference in ionic radii of substitution partners is <15%.^33^ The ionic radii of I^−^ and Br^−^ are 220 and 196 pm, respectively, which leads to a difference of 18%, thus a full solubility cannot be expected.^34^ Samples with overall composition within a miscibility gap are expected to contain an iodine rich and a bromine rich phase of MAPb(I_1−*x*_Br_*x*_)_3_. This can be easily identified by the appearance of additional Bragg peaks in the powder diffraction pattern. For instance Fig. S1 (ESI)† shows the Le Bail refinement of a powder pattern of MAPb(I_0.83_Br_0.17_)_3_ solid solution as main phase, with LaB_6_ as internal standard. The Fig. 1 shows the evolution of the 220 and 004 Bragg peak with increasing Br amount. Single phase peaks are visible at *x* < 0.29 (±0.02) and *x* > 0.92 (±0.02). Two clearly separated peaks are visible between 0.29 (±0.02) ≤ *x* ≤ 0.92 (±0.02), corresponding to two phases. In the following we will refer to the phase with larger lattice parameters as “iodine rich phase”, to the one with smaller lattice parameters as “bromine rich phase”. As we will show in the following discussion, these denominations are in good agreement with the observed results.

On the contrary to our results, a study on the solubility of the MAPb(I_1−*x*_Br_*x*_)_3_ series published before, shows a full solubility on thin film samples.^11^ Other groups^35^ predict a thermodynamic metastability of this solid solution for intermediate Br/I compositions from DFT calculations, confirming our results of the existence of a large two phase region. While studies on the MAPb(I_1−*x*_Cl_*x*_)_3_ series revealed a large miscibility gap,^36^ where the lattice parameters would show a clustering at the edges of miscibility and the formation of two separated phases with nearly constant lattice parameter for each phase within the gap, for the MAPb(I_1−*x*_Br_*x*_)_3_ series a more complicated behavior is observed. Even though a clear phase separation is visible in the miscibility gap, the lattice parameter values do not remain constant. In contrast to the expected behavior of lattice parameters within a miscibility gap the lattice parameters of the phases decrease with increasing overall bromine amount. Furthermore, Le Bail and Rietveld refinement required the use of the tetragonal model (*I*4/*mcm*) for the iodine rich phase over the whole composition range. With the cubic model the strong *hkl*-dependent asymmetries of the Bragg peaks could not be fitted. This is in strong contrast to the observed absence of tetragonal super lattice reflections for samples with *x* ≥ 0.23. In addition, for these samples the cubic to tetragonal phase transition is below room temperature (see sub-Section 3.3), they thus should crystalize in the cubic space group (*Pm*3̄*m*). In contrast, the bromine rich phase could always be fitted using cubic *Pm*3̄*m*.

### 3.1 Lattice parameters at ambient conditions

Assuming a complete solid solution, the change of lattice parameter can be described by an approximate empirical rule, well known as Vegard's law. This law states that in an ideal solution the lattice parameters (at constant temperature) vary linearly with the concentration of constituent elements.^37^ Therefore, a simple mathematical expression for a binary solid solution A–B with *x* = *x*_B_ for the mole fraction of component B, *a*^0^_A_ and *a*^0^_B_ for the lattice parameters of pure components A and B can be described as follows:*a* = *a*^0^_A_(1 − *x*) + *a*^0^_B_(*x*).

Since MAPbI_3_ crystalizes in the tetragonal space group *I*4/*mcm* at ambient conditions,^18^ whereas MAPbBr_3_ crystalizes in the cubic space group *Pm*3̄*m*,^38^ the tetragonal lattice parameter *a*_tet_ and *c*_tet_ of MAPbI_3_ were converted to the pseudo-cubic lattice parameter *a*_ps-cub_, using the following equations:
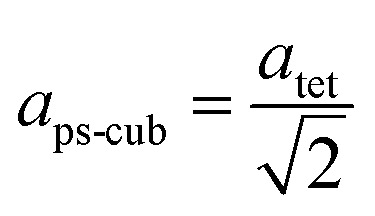

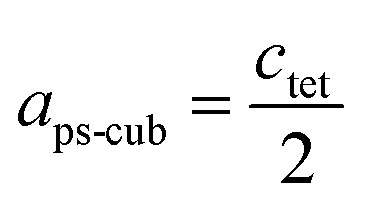


**Fig. 2:**
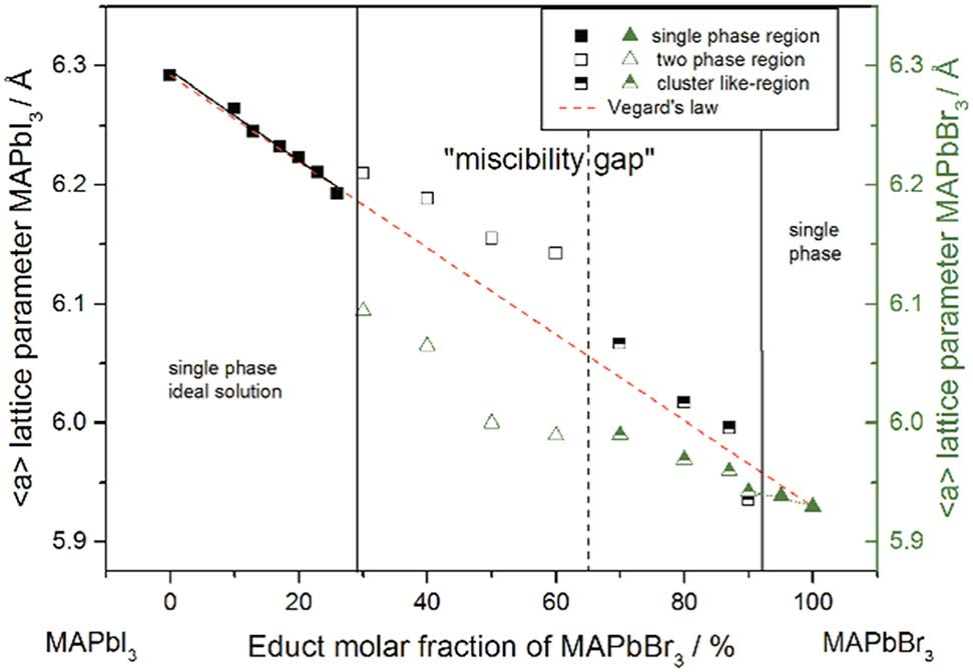
Room temperature cubic mean lattice parameter 〈*a*〉 of MAPb(I_1−*x*_Br_*x*_)_3_ solid solutions and Vegard derived linear interpolation of cubic lattice parameters (dashed): lattice parameters of the iodine rich part follow Vegard's law whereas lattice parameters in the two-phase region do not show a linear trend. The miscibility gap is divided into two regions: empty squares and triangles corresponding to clearly resolved iodine rich and bromine rich peaks, half-filled squares and triangles corresponding to bromine rich peaks with iodine cluster-like regions.

For a better comparability an average lattice parameter 〈*a*〉 was calculated from both *a*_ps-cub_ lattice parameters. These mean lattice parameters of each single phase sample and Vegard derived linear interpolation of cubic lattice parameters in dependence of educt molar fraction are shown in Fig. 2.

Note that, for all samples the composition of the phase is obviously the same as the weighing without further analysis. In Fig. 2 the dependence of the room temperature cubic mean lattice parameter 〈*a*〉 of MAPb(I_1−*x*_Br_*x*_)_3_ solid solutions in dependence of educt molar fraction of MAPbBr_3_ is shown. To compare the experimental results with the behavior in an ideal solid solution, a Vegard derived linear interpolation of cubic lattice parameters is shown as well. Single phase regions are displayed in filled squares and triangles between *x* < 0.29 (±0.02) and *x* < 0.92 (±0.02). The region of miscibility gap is visible between 0.29 (±0.02) ≤ *x* ≤ 0.92 (±0.02), corresponding to an iodine rich and a bromine rich phase. Further, differentiating between a two-phase region (a) and a cluster-like region (b) to clearly describe the miscibility gap has been done and will be discussed.

The pseudo cubic lattice parameters of the iodine rich single phase samples (0 ≤ *x* ≤ 0.29) (black filled squares) and the cubic lattice parameters of the bromine rich single phase samples (0.92 ≤ *x* ≤ 1.0) (green filled triangles) show a decreasing linear trend in their region of solubility, which was observed in other studies as well.^26^ A fit of the iodine rich single phase region results in 〈*a*〉 = 6.292(1)Å − 0.0038(2)Å/% *x*.

In an ideal solution Vegard's law would predict a linear dependency of the lattice parameters from the composition, which could be described by linear interpolation between the pure end members. This would result in a slope of −0.0036 Å/%. Comparing this to the slope of the experimental results, the lattice parameters of the iodine rich single phase samples are in good agreement with the trend predicted by Vegard's law. On the other hand, the bromine rich single phase region is extensively smaller, with only two single phase samples synthesized (green triangles in the right part of Fig. 2). This does not allow for a compulsive linear interpolation. The trend for the lattice parameters of the bromine rich samples (0.90 < *x* < 1), marked with a dotted line, however, indicates a lower slope as expected from Vegard's law, which extends far into the miscibility gap for the bromine rich phase. This can be explained by the crystal structure being stiffer than needed for ideal solution behavior. The coordinating cations do not adapt perfectly to the change of the anion size, but are held back by interactions with the rest of the framework. Consequently, calculating the composition from the lattice parameters of any given MAPb(I_1−*x*_Br_*x*_)_3_ phase within the two-phase region might lead to wrong results, if they are based on an assumption of Vegard's law. This has implications in particular for technologically relevant studies on thin films, where such an approach is often taken, as for example in the work of Noh *et al.*^11^

### 3.2 The nature of the miscibility gap

Although the Le Bail refinement results show a constant decrease of lattice parameters in the two phase region (from 0.29 (±0.02) ≤ *x* ≤ 0.92 (±0.02)), this region must be split in two parts: (a) the compositions, showing two clearly separated iodine rich and bromine rich peaks (0.29 (±0.02) ≤ *x* ≤ 0.65 (±0.05)) and (b) the part, with only one Bragg peak visible, showing an asymmetric peak shape (0.65 (±0.05) ≤ *x* ≤ 0.92 (±0.02)), where the iodine rich phase is insufficiently resolved, but the bromine rich phase is strongly pronounced.

Rietveld analysis allowed refining the iodine to bromine ratio of all phases as well as their weight fractions (ESI: Table S2, Fig. S2†). Calculating from this the total composition of the sample, the results are in good agreement with the initial weights. This is a very strong indicator for the reliability of the individual results from the Rietveld refinements.

**Fig. 3:**
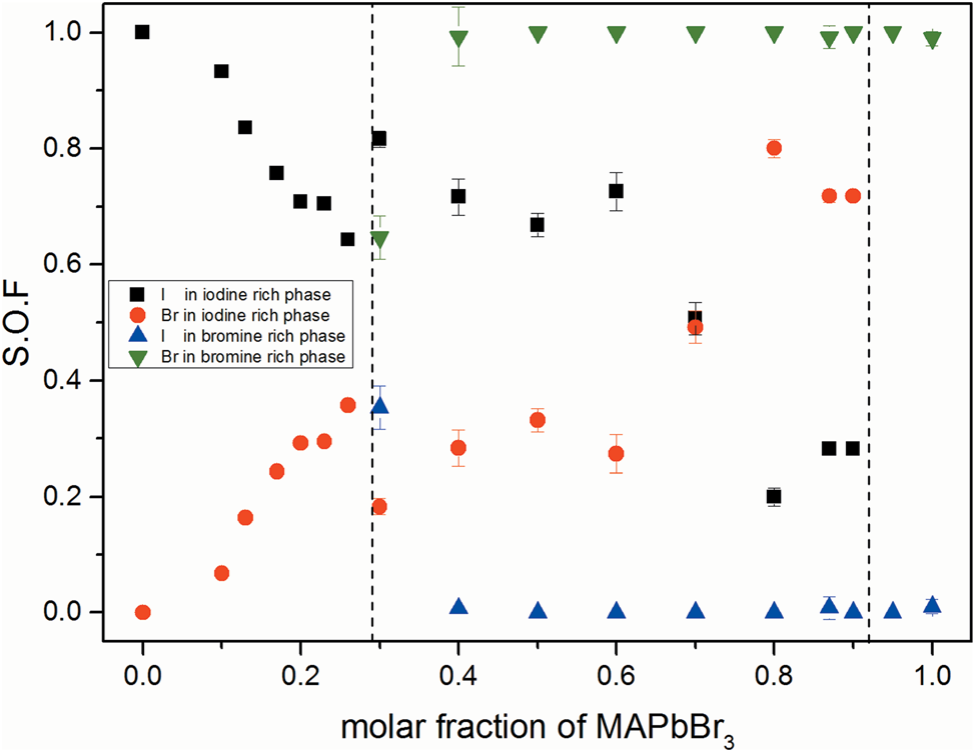
Site occupancy factors in dependence of educt molar fraction of MAPb(I_1−*x*_Br_*x*_)_3_ received from Rietveld refinement of XRD powder pattern. Occupancy factors of iodine rich and bromine rich phases in single phase and miscibility gap are shown. For a better comparability, the two phase region is separated by horizontal dotted lines.

Fig. 3 shows the iodine/bromine site occupancy factors for all overall compositions. The iodine rich single phase region shows a nearly linear decrease of the iodine site occupancy factors (S.O.F.) with increasing bromine content. This is in good agreement with the linear dependency of the lattice parameters, following Vegard's law. In this composition range the incorporation of bromine into the crystal structure of MAPbI_3_ happens as an ideal solid solution.

In the two phase region (0.29 (±0.02) ≤ *x* ≤ 0.92 (±0.02)) the iodine rich phase is increasingly incorporating bromine, over the whole composition range up to 80%. Simultaneously, however, the fraction of this phase decreases. Instead increasing amounts of the bromine rich phase form. No substitution of bromine with iodine could be observed, the bromine rich phase refines as pure MAPbBr_3_. There is some uncertainty about the accuracy of this absolute purity. The site occupation ratio is correlated with the Debye–Waller factors of the anion which was not refined. For *x* > 0.92 no second phase could be observed, indicating that up to 8% iodine can be incorporated into the structure of the MAPbBr_3_ phase. The behavior of the phase transitions (sub-Section 3.3), in particular the vanishing of the IC-phase and the small decrease of the transition temperatures, seems to corroborate this interpretation.

Surprisingly, the lattice parameter of the bromine rich phase nonetheless increases with the iodine content of the overall sample. This can be explained by a strong intergrowth of iodine and bromine rich crystals in the coexistence region. Due to this intergrowth the lattice parameters of both phases cannot relax to their ideal values. Such an intergrowth can be expected to give rise to strong peak broadening and asymmetry, just as observed within the coexistence region. This effects mostly the iodine rich phase, which undergoes actual I–Br substitution.

The observed behavior differs from a classical miscibility gap. The possible composition range of one of the two phases extends over large parts of the gap, albeit in decreasing weight fractions. In addition, the two phases strongly interact. This interaction is particularly strong in what we labeled the cluster-like region (Fig. 1), where the two phases are chemically very similar. However, in the range 0.29 ≤ *x* ≤ 0.62 the existence of a miscibility gap is obvious. This has strong implications on the behavior of mixed halide perovskite systems, where this phase separation can directly affect the electronic properties of the compounds.

This presence of a miscibility gap is in strong contrast to the results published by Noh *et al.*, who shows a complete solubility behavior.^11^ Based on diffraction data, they show only one single peak. The FWHM of the Bragg peaks corresponding to the tetragonal 004 and 220 as well as the cubic 200 reflections reveal a broadening of the peaks between 0.20 < *x* < 0.58, which is in contrast to the sharp peak width of single phase samples (*i.e. x* = 1). In addition, the peak shape is considerable asymmetric, indicating that there are unresolved crystalline phases with varying degrees of substitution. On top of that the sample *x* = 0.29 shows a clearly visible shoulder, which supports the above statement. The missing clear peak separation might be due to the crystallization conditions, causing a lower crystallinity of thin film samples and thus differing from powder material presented in this work.

### 3.3 Temperature dependency of lattice parameters

Using *in situ* synchrotron X-ray diffraction temperature dependent changes of the lattice parameters of different MAPb(I_1−*x*_Br_*x*_)_3_ solid solution members have been investigated. The thermal expansion of the unit cell is shown for the end members, MAPbI_3_ and MAPbBr_3_, and exemplarily for MAPb(I_0.8_Br_0.2_)_3_ as a representative composition of iodine rich single phase region between 0 ≤ *x* < 0.29.

**Fig. 4:**
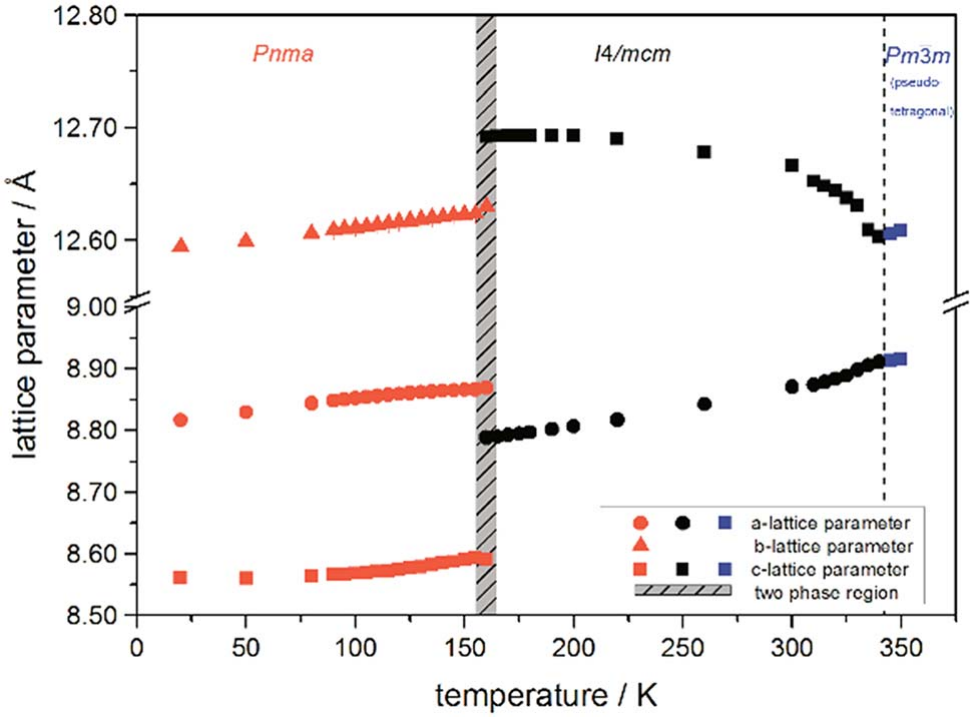
Temperature dependent lattice parameters of MAPbI_3_ received from Le Bail refinement of synchrotron XRD pattern. Lattice parameters of orthorhombic, tetragonal and cubic phase are shown. For a better comparability, the pseudo tetragonal *a*- and *c*-lattice parameters are used in the high temperature cubic (*Pm*3̄*m*) modification at the phase transition to the lower temperature tetragonal phase.

**Fig. 5:**
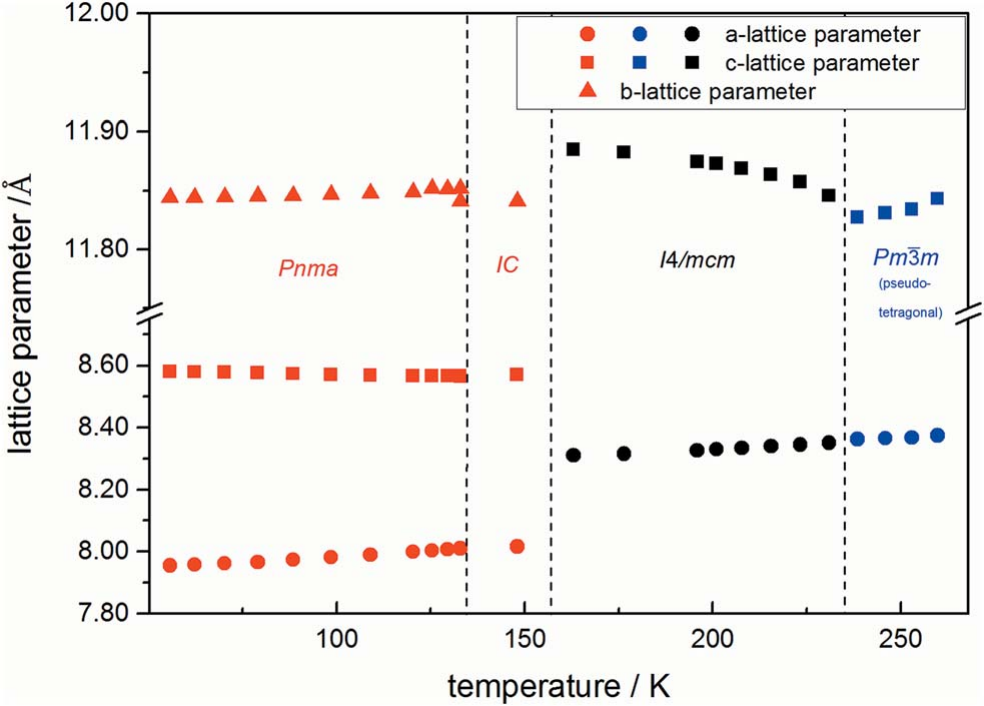
Temperature dependent lattice parameters of pure MAPbBr_3_ received from Le Bail refinement of synchrotron XRD pattern. Lattice parameters of orthorhombic, tetragonal and cubic phase are shown. For a better comparability, the pseudo tetragonal *a*- and *c*-lattice parameters are used in the high temperature cubic (*Pm*3̄*m*) modification at the phase transition to the lower temperature tetragonal phase. In addition an incommensurate phase (IC) is present.

The change in unit cell volume of all three components over the complete temperature range was compared. In MAPbI_3_ (Fig. 4) and MAPbBr_3_ (Fig. 5) a linear thermal expansion of the unit cell with a continuous increase of the lattice parameters can be seen over the complete temperature range.

**Fig. 6:**
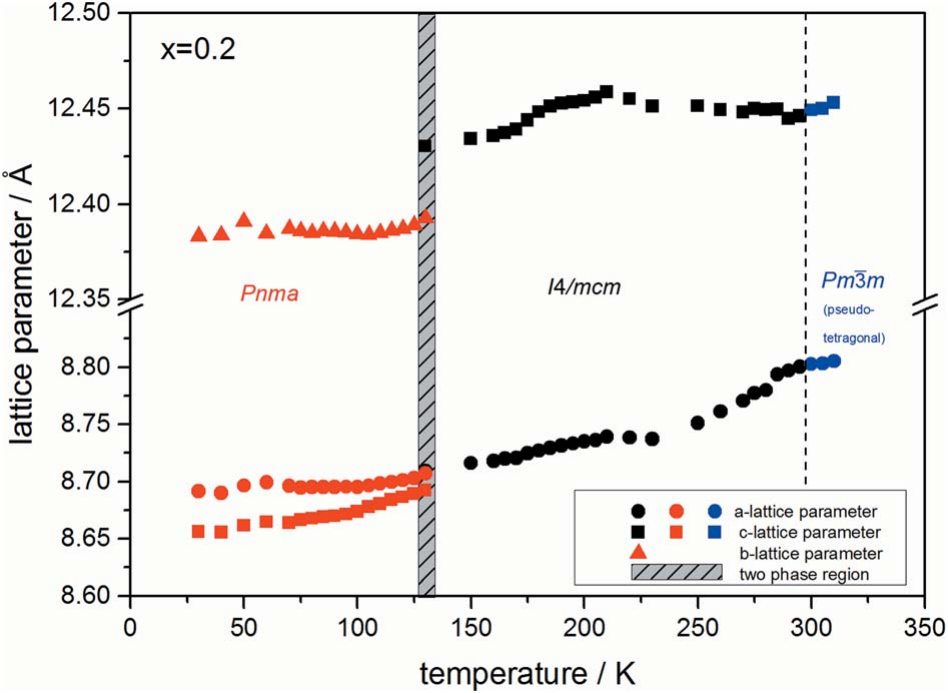
Temperature dependent lattice parameters of MAPb(I_0.8_Br_0.2_)_3_ solid solution received from Le Bail refinement of synchrotron XRD pattern. Lattice parameters of orthorhombic, tetragonal and cubic phase are shown. For a better comparability, the pseudo tetragonal *a*- and *c*-lattice parameters are used in the high temperature cubic (*Pm*3̄*m*) modification at the phase transition to the lower temperature tetragonal phase.

At the orthorhombic (*Pnma*) to tetragonal (*I*4/*mcm*) phase transition a discontinuous change of the unit cell volume is present, which gives evidence for a first-order character of this phase transition. The phase transition from tetragonal (*I*4/*mcm*) to cubic (*Pm*3̄*m*) is of a second-order character, as shown by other groups as well.^24,39^ Additionally, in MAPbBr_3_ an incommensurate phase (IC) is present in the temperature range between 148 and 154 K, which will not be further investigated in this work. For the MAPb(I_0.8_Br_0.2_)_3_ solid solution (Fig. 6) the orthorhombic–tetragonal phase transition is of first order and the tetragonal–cubic phase transition shows a second order phase transition as well. In contrast to the pure MAPbI_3_ and MAPbBr_3_ phase, in which a linear thermal behavior of *a* and *c* lattice parameters can be observed close to the orthorhombic–tetragonal phase transition, the solid solution shows an alignment of *a*- and *c*-lattice parameter values. Further, a strong discontinuous increase of the *b*-lattice parameter is observed, meaning that the volume leap is mainly independent of the *a*-lattice and *c*-lattice parameters in the low temperature phase.

**Fig. 7:**
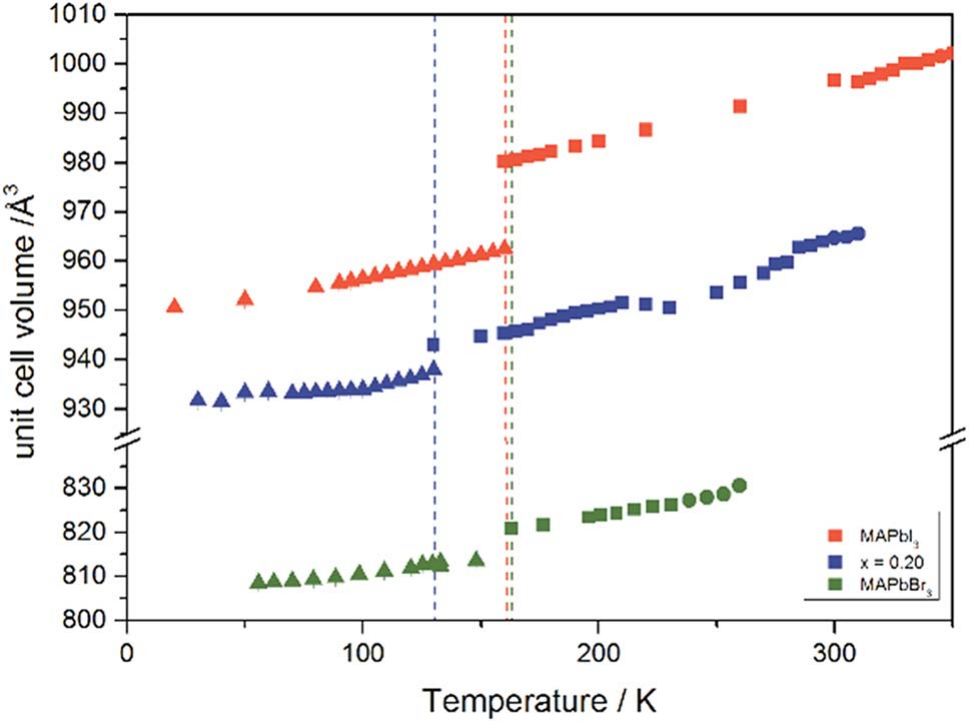
Temperature dependence of the unit cell volume for MAPbI_3_, MAPbBr_3_ and MAPb(I_1−*x*_Br_*x*_)_3_ solid solutions with *x* = 0.20.

The Fig. 7 illustrates the evolution of the unit cell volume over the temperature range of 0 to 350 K for MAPbI_3_, MAPbBr_3_ and for the MAPb(I_0.8_Br_0.2_)_3_ solid solution to show the effect of substitution on the unit cell volume. For MAPbI_3_ and MAPbBr_3_ a linear thermal expansion of the unit cell volume can be seen over the complete temperature range. In the MAPb(I_0.8_Br_0.2_)_3_ solid solution the thermal expansion of the unit cell volume is different: close to the orthorhombic to tetragonal phase transition temperature the unit cell volume shows a strong increase due to the alignment of the *a*-lattice and *c*-lattice parameters. Comparing the results it can be seen, that the volume leap at the phase transition in the solid solution is far smaller than in the pure MAPbI_3_ endmember. This might be related to the incorporation of the smaller bromine in the crystal structure, creating a lower energy barrier for the stretching of the Pb–I-bonding.

**Fig. 8:**
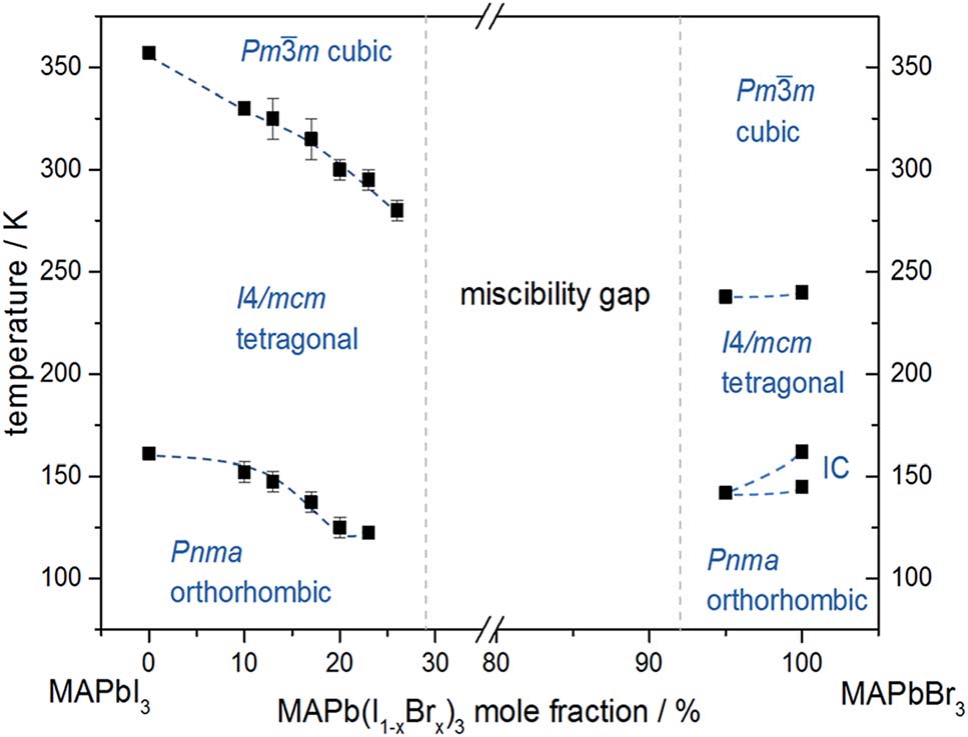
Phase diagram of MAPb(I_1−*x*_Br_*x*_)_3_ solid solution showing the phase transition temperatures over a temperature range of 30 to 350 K.

*In situ* temperature dependent X-ray diffraction experiments provide information about structural phase transitions in the MAPb(I_1−*x*_Br_*x*_)_3_ solid solution series, allowing to establish a complete *T*–*X* phase diagram (Fig. 8).

The general trend shows that the phase transition temperatures decrease with increasing bromine-amount in iodine rich compositions of the solid solution series (*x* < 0.29 (±0.02)). A strong decrease of the transition temperature is visible for the tetragonal to cubic phase transition. Thus the incorporation of bromine in the crystal structure leads to an extended sustainability of the full-disordered state of the organic cation and stabilizes the cubic high temperature phase. A slight decrease of the tetragonal to orthorhombic phase transition temperatures is visible, meaning that the substitution of iodine by bromine increases the disorder in the crystal lattice. On the other hand, the phase transition temperatures on the bromine rich side (*x* > 0.92 (±0.02)) are less affected. The cubic to tetragonal and tetragonal to orthorhombic phase transition temperatures show a maximum decrease of ±1 K.

By assuming that the incorporation of iodine in the MAPbBr_3_ structure leads to an elongation of the Pb–Br-bond lengths and an increase in lattice parameter, the reorientation of the organic cation should be sustained. Surprisingly, the bromine rich compositions behave contradictious. This could be related to strong intermolecular forces, in specific hydrogen bonding, between the N–H bond of the molecule and the (N–H–I), stabilizing the order in the crystal structure, an thus suppressing the reorientation of the cation. Comparing the acidity of H–Br (p*K*_s_ = −9) with H–I (p*K*_s_ = −11) it is obvious, that bromine creates a stronger hydrogen bond to the N–H bond of the organic cation. Hence, the incorporation of iodine does not affect the MAPbBr_3_ structure, which is as well confirmed by the nearly constant phase transition temperature of the cubic to tetragonal phase for bromine rich compositions. Comparing this behavior to the tetragonal to orthorhombic phase transition temperatures, the substitution of bromine by iodine seems to destabilize the order in the structure, thus the phase transition temperature decreases.

An interesting result, arising in MAPbBr_3_ only, is the existence of an incommensurate phase (IC) between 148 and 154 K, which does not appear in the solid solution.

Until now, this phase has been reported a few times, but has not been described sufficiently.^18,40^ A possible mechanism for the non-existence of the IC phase in the solid solution, could be the influence of the iodine clusters occurring with already very low iodine content (*x* < 0.1) in MAPbBr_3_.

These results show that the phase transition temperatures are highly affected by the substitution of iodine by the smaller Br-anion in MAPbI_3_, while the substitution of bromine by the larger iodine in MAPbBr_3_ is less influenced.

### 3.4 Photoluminescence

**Fig. 9:**
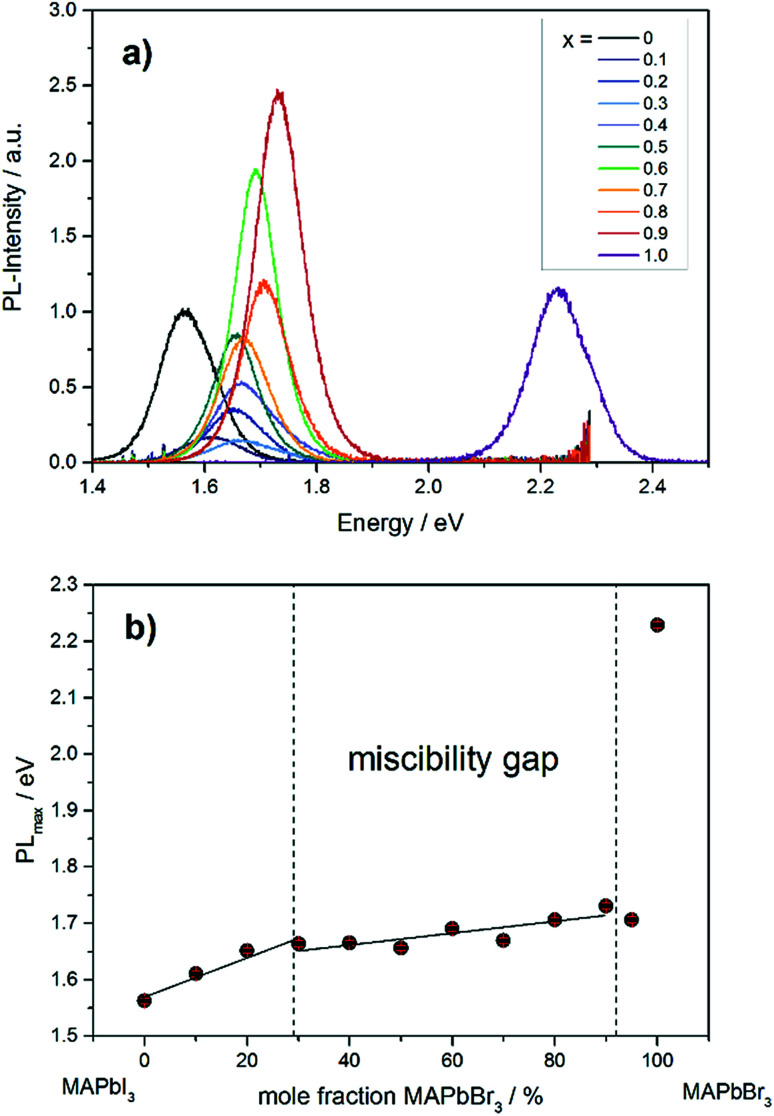
(a) Photoluminescence spectra of different MAPb(I_1−*x*_Br_*x*_)_3_ mixed crystals. (b) Dependence of PL_max_ on the chemical composition in MAPb(I_1−*x*_Br_*x*_)_3_ solid solutions.

The photoluminescence spectra in Fig. 9a show the variation of band gap energy in dependence of the chemical composition.

The PL_max_ of MAPbI_3_ can be located at 1.56 eV, while for MAPbBr_3_ the maximum intensity is detected at 2.23 eV. The PL_max_ is shifted to higher energies with increasing bromide content in MAPb(I_1−*x*_Br_*x*_)_3_. For comparison, the MAPb(I_1−*x*_Br_*x*_)_3_ solid solutions show PL_max_ values varying between 1.60 and 1.73 eV, which is in good agreement with previous reported results.^41^ By taking the maximum PL-intensity of each composition and plotting these values in dependence of chemical composition, the bandgap variation of the solid solution can be revealed, shown in Fig. 9b. Within the single phase region (up to *x* = 0.29 (±0.02)) a linear increase of the PL_max_ is visible. In the two-phase region (0.29 (±0.02) ≤ *x* ≤ 0.92 (±0.02)) the bandgap value remains nearly constant, varying between 1.6 to 1.73 eV, which confirms the presence of phase segregation, forming iodine rich and bromine rich phases. Within the cubic single phase region (above *x* = 0.92 (±0.02)) a linear trend could not be observed.

## 4. Conclusions

The MAPb(I_1−*x*_Br_*x*_)_3_ solid solution series has been investigated in terms of solubility and structural phase transitions over a temperature range of 30 to 350 K. X-ray powder diffraction revealed that MAPb(I_1−*x*_Br_*x*_)_3_ solid solution forms a two phase region between 0.29 (±0.02) ≤ *x* ≤ 0.92 (±0.02). Furthermore, by analyzing the lattice parameters obtained by Le Bail refinement of the X-ray powder diffraction pattern, a linear dependency of the chemical composition on the iodine rich mixed crystals of the MAPb(I_1−*x*_Br_*x*_)_3_ solid solution series was revealed, showing a behavior according to Vegard's law. In contrast, the lattice parameters of the bromine rich mixed crystals do not follow Vegard's law. This is likely due to the large difference in ionic radii of iodine and bromine. In the miscibility gap the bromine rich compositions do show Bragg peaks with asymmetric shape, indicating an apparent iodine cluster formation in the range of 0.65 (±0.05) ≤ *x* ≤ 0.92 (±0.02). It needs to be mentioned, that a continuous shift of lattice parameter for the iodine rich compositions occurs in the two phase region, indicating a partial solubility of bromine in iodine crystals, while no substitution of iodine in bromine rich phase was observed. Moreover, using *in situ* high temperature and low temperature synchrotron X-ray diffraction, the temperature dependent phase diagram of the MAPb(I_1−*x*_Br_*x*_)_3_ solid solution as well as temperature dependency of the lattice parameters were established. For the iodine rich mixed crystals phase transition temperatures follow a decreasing trend, due to the increasing iodine-bromine substitution. This leads to increased disorder in the crystal structure and stabilizes the cubic high temperature phase. The phase transition temperatures of the bromine rich compositions seem to be unaffected by the iodine substitution. Complementary optoelectronic measurements were performed, using photoluminescence spectroscopy, revealing a tunable bandgap from 1.56 to 1.66 eV in dependence of halide composition in the range of *x* < 0.29 (±0.02). For compositions within the miscibility gap the bandgap value stays nearly constant, confirming the existence of a coexistence region. These results lead to a fundamental understanding of the influence on structure and transition temperatures of halide substitution in hybrid perovskite MAPb(I_1−*x*_Br_*x*_)_3_ solid solution.

## Conflicts of interest

There are no conflicts to declare.

## Supplementary Material

RA-009-C8RA09398A-s001
