# Supplementary material for: The phase diagram of a mixed halide (Br, I) hybrid perovskite obtained by synchrotron X-ray diffraction
Source: RSC Adv. 2019 Apr 9;9(20):11151–9. doi: 10.1039/c8ra09398a (PMC9063018; doi:10.1039/c8ra09398a)
Supplement: RA-009-C8RA09398A-s001 [file RA-009-C8RA09398A-s001.pdf]

Electronic Supplementary Material (ESI) for RSC Advances.
This journal is © The Royal Society of Chemistry 2019

The phase diagram of mixed halide (Br, I) hybrid perovskites obtained by synchrotron X-ray diffraction

Frederike Lehmann[ab] Alexandra Franz [a] Daniel M. Többens[a] Sergej Levcenco[a] Thomas Unold[a] Andreas Taubert[b] and Susan Schorr[ac]*

[a]Helmholtz-Zentrum Berlin für Materialien und Energie, Hahn-Meitner Platz 1, 14109 Berlin, Germany.

[b]University of Potsdam, Institute of Chemistry, Karl-Liebknecht-Straße 24-25, 14476 Potsdam OT Golm, Germany

[c]Free University Berlin, Department of Geoscience, Malteserstr. 74-100, 12249 Berlin, Germany

*Corresponding author, email: susan.schorr@helmholtz-berlin.de

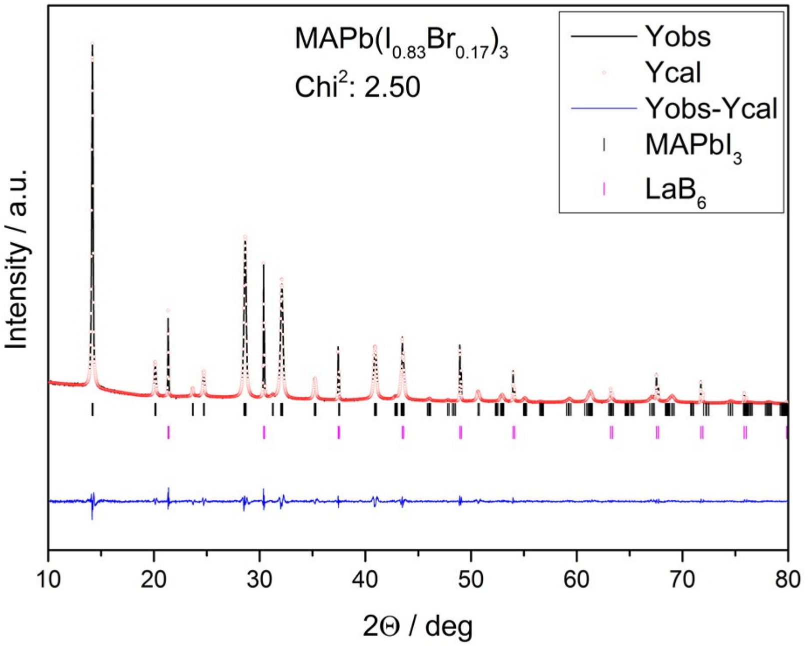

Figure S1| Le Bail refinement of the powder pattern of the $MAPb(I_{0.83}Br_{0.17})_3$ solid solution

Table S1|Total composition, lattice parameters (tetragonal for iodine rich compositions; cubic for bromine rich compositions) and single or multiple phases

Total composition	single/ multiple phases	Pseudo cubic <a> lattice parameter [Å]	Tetragonal a lattice parameter [Å]	Tetragonal c lattice parameter [Å]	Cubic a lattice parameter [Å]
$MAPbI_3$	single	6.292 ± 0.001	8.8691(06)	12.6675(1)	-
$MAPb(I_{0.9}Br_{0.1})_3$	single	6.264 ± 0.001	8.8395(09)	12.5847(2)	-
$MAPb(I_{0.87}Br_{0.13})_3$	single	6.245 ± 0.001	8.8188(2)	12.5247(4)	-
$MAPb(I_{0.83}Br_{0.17})_3$	single	6.232 ± 0.001	8.8051(2)	12.4903(4)	-
$MAPb(I_{0.8}Br_{0.2})_3$	single	6.223 ± 0.001	8.7991(3)	12.4518(4)	-
$MAPb(I_{0.77}Br_{0.23})_3$	single	6.211 ± 0.001	8.7837(2)	12.4194(1)	-
$MAPb(I_{0.74}Br_{0.26})_3$	single	6.192 ± 0.001	8.7590(2)	12.3820(3)	-
$MAPb(I_{0.7}Br_{0.3})_3$	multiple	6.209 ± 0.01	8.8122(2)	12.3318(7)	6.0939(3)
$MAPb(I_{0.6}Br_{0.4})_3$	multiple	6.188 ± 0.01	8.7680(05)	12.3286(2)	6.0645(3)
$MAPb(I_{0.5}Br_{0.5})_3$	multiple	6.155 ± 0.01	8.7544(4)	12.1681(13)	5.9995(2)
$MAPb(I_{0.4}Br_{0.6})_3$	multiple	6.147 ± 0.01	8.7477(4)	12.1411(7)	5.9957(2)
$MAPb(I_{0.3}Br_{0.7})_3$	multiple	6.067 ± 0.01	8.6017(10)	12.0702(16)	5.9898(2)
$MAPb(I_{0.2}Br_{0.8})_3$	multiple	6.017 ± 0.01	8.5115(20)	12.0250(12)	5.9690(1)
$MAPb(I_{0.13}Br_{0.87})_3$	multiple	5.995 ± 0.01	8.4815(6)	11.9833(10)	5.9596(07)
$MAPb(I_{0.1}Br_{0.9})_3$	multiple	5.935 ± 0.01	8.3914(1)	11.8781(12)	5.9420(06)
$MAPb(I_{0.05}Br_{0.95})_3$	single	-	-	-	5.9385(04)
$MAPbBr_3$	single	-	-	-	5.9293 (02)

Table S2| Total composition, weight fractions of iodine rich phase (Both phase compositions of iodine an bromine at up to 100% in all cases.) and site occupancy factors (S.O.F.) for bromine in iodine rich- and bromine rich phase

Total composition	weight fraction iodine rich phase %	S.O.F. bromine in iodine rich phase	Error S.O.F. bromine in iodine rich phase	S.O.F. bromine in bromine rich phase	Error S.O.F. bromine in bromine rich phase
$MAPbI_3$	100.00 (0.81)	0.000	-	-	-
$MAPb(I_{0.9}Br_{0.1})_3$	100.00 (0.79)	0.068	0.007	-	-
$MAPb(I_{0.87}Br_{0.13})_3$	100.00 (0.69)	0.164	0.007	-	-
$MAPb(I_{0.83}Br_{0.17})_3$	100.00 (0.64)	0.243	0.006	-	-
$MAPb(I_{0.8}Br_{0.2})_3$	100.00 (0.70)	0.292	0.007	-	-
$MAPb(I_{0.77}Br_{0.23})_3$	100.00 (0.66)	0.295	0.005	-	-
$MAPb(I_{0.74}Br_{0.26})_3$	100.00 (0.57)	0.358	0.005	-	-
$MAPb(I_{0.7}Br_{0.3})_3$	71.53 (1.85)	0.183	0.014	0.647	0.037
$MAPb(I_{0.6}Br_{0.4})_3$	82.15 (2.76)	0.283	0.031	0.993	0.051
$MAPb(I_{0.5}Br_{0.5})_3$	73.27 (1.41)	0.332	0.020	1.000	-
$MAPb(I_{0.4}Br_{0.6})_3$	73.62 (2.37)	0.274	0.033	1.000	-
$MAPb(I_{0.3}Br_{0.7})_3$	65.34 (2.82)	0.492	0.028	1.000	-
$MAPb(I_{0.2}Br_{0.8})_3$	46.57 (1.57)	0.801	0.016	1.000	-
$MAPb(I_{0.13}Br_{0.87})_3$	63.26 (1.39)	0.718	0.011	0.992	0.019
$MAPb(I_{0.1}Br_{0.9})_3$	20.15 (1.83)	0.718	0.000	1.000	-
$MAPb(I_{0.05}Br_{0.95})_3$	0.00	0.000	-	1.000	-
$MAPbBr_3$	0.00	0..000	-	0.990	0.012

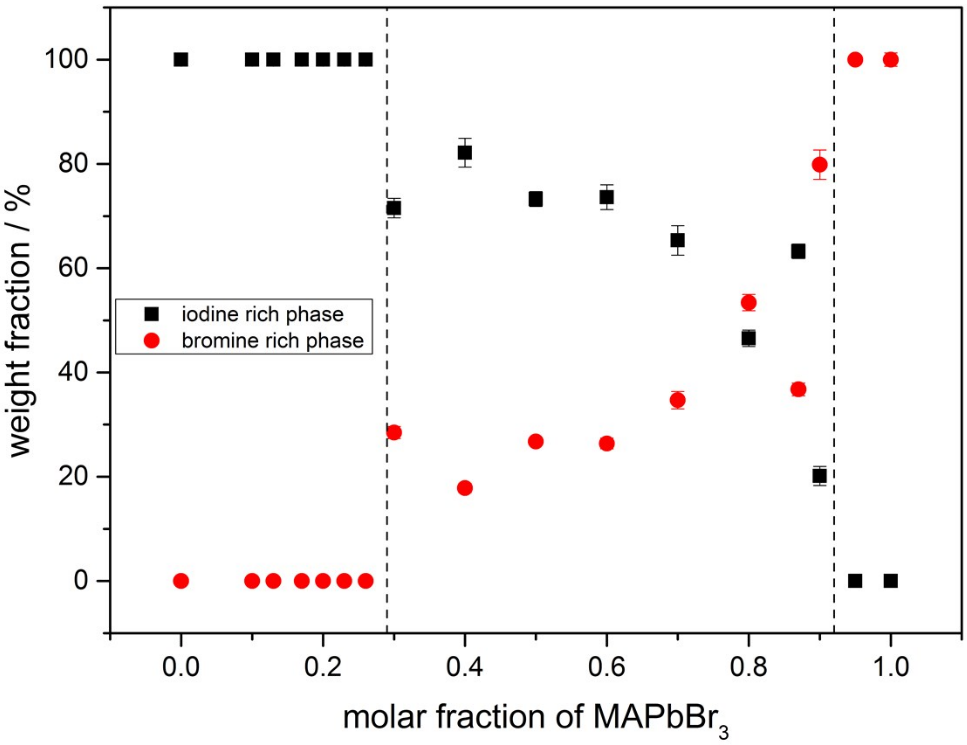

Figure S2 | Weight fractions of the iodine and bromine rich phases received by Rietveld refinement of the powder pattern of the $MAPb(I_{1-x}Br_x)_3$ solid solution (black rectangles for iodine rich phase, red circles for bromine rich phase)

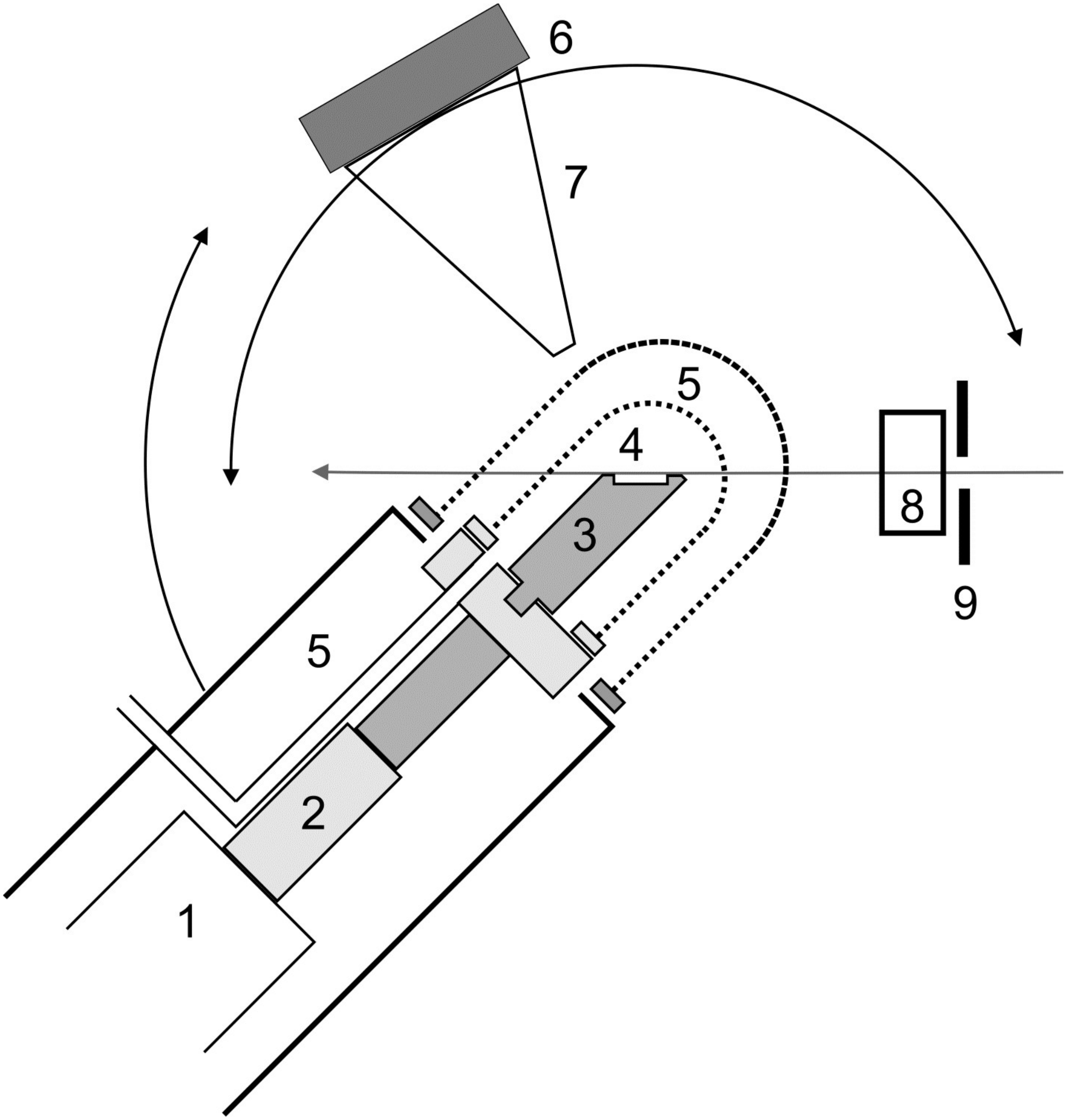

Figure S3| Sketch of the low temperature synchrotron X-ray diffraction setup at the KMC-2 beamline (BESSY II) (Gifford-McMahon closed-cycle cryo-cooler (1) equipped with additional high temperature stage (2), sample holder (3) for reflection in He-exchange gas cell (4), with double Kapton dome providing isolation vacuum (5). Mounted in Eulerian cradle of KMC-2 goniometer (not to scale), with Vantec 2000 area detector (6) with anti-scattering cone (7), monitor counter (8) and primary beam slits (9) indicated. Operated in symmetric reflection geometry.) [1]

References:

[1] V. Bon *et a*l., Microporous and Mesoporous Materials 188 (2014) 190–195.
